# G-749 Promotes Receptor Tyrosine Kinase TYRO3 Degradation and Induces Apoptosis in Both Colon Cancer Cell Lines and Xenograft Mouse Models

**DOI:** 10.3389/fphar.2021.730241

**Published:** 2021-10-14

**Authors:** Hae Dong Kim, Eun Jung Park, Eun Kyoung Choi, Seuk Young Song, Kwang-Lae Hoe, Dong-Uk Kim

**Affiliations:** ^1^ Rare Disease Research Center, Korea Research Institute of Bioscience and Biotechnology (KRIBB), Daejeon, South Korea; ^2^ Department of New Drug Development, Chungnam National University, Daejeon, South Korea; ^3^ Application Strategy and Development Division, GeneChem Inc., Daejeon, South Korea

**Keywords:** G-749, anticancer drug, TAM receptor tyrosine kinase, tyro3, RIP process, protein degradation

## Abstract

G-749 is an FLT3 kinase inhibitor that was originally developed as a treatment for acute myeloid leukemia. Some FLT3 kinase inhibitors are dual kinase inhibitors that inhibit the TAM (Tyro3, Axl, Mer) receptor tyrosine kinase family and are used to treat solid cancers such as non-small cell lung cancer (NSCLC) and triple-negative breast cancer (TNBC). AXL promotes metastasis, suppression of immune response, and drug resistance in NSCLC and TNBC. G-749, a potential TAM receptor tyrosine kinase inhibitor, and its derivative SKI-G-801, effectively inhibits the phosphorylation of AXL at nanomolar concentration (IC_50_ = 20 nM). This study aimed to investigate the anticancer effects of G-749 targeting the TAM receptor tyrosine kinase in colon cancer. Here, we demonstrate the potential of G-749 to effectively inhibit tumorigenesis by degrading TYRO3 via regulated intramembrane proteolysis both *in vitro* and *in vivo*. In addition, we demonstrated that G-749 inhibits the signaling pathway associated with cell proliferation in colon cancer cell lines HCT15 and SW620, as well as tumor xenograft mouse models. We propose G-749 as a new therapeutic agent for the treatment of colon cancer caused by abnormal TYRO3 expression or activity.

## Introduction

G-749 is an FLT3 kinase (FMS-like tyrosine kinase 3, FLT3) inhibitor, which was originally developed as a treatment for acute myeloid leukemia (AML) and later designated as an orphan drug by the Federal Drug Administration (FDA) in 2018. The IC_50_ values for FLT3 kinase of the small molecule inhibitor G-749, synthesized using a structure-based drug design approach, were reported to be 0.4 and 0.6 nM for WT-FLT3 kinase and D835Y-FLT3 mutants, respectively. G-749 and its derivative SKI-G-801, which is an ATP-competitive inhibitor, showed strong inhibitory activity against FLT3 kinase with low nanomolar IC_50_ values (0.4–2.7 nM) even at 1 mM ATP. A kinase inhibition assay using the Millipore Kinase Profiler revealed that G-749 exhibited strong inhibition of MER, Aurora and AXL at nanomolar IC_50_ values (1, 6, and 20 nM, respectively). MER and AXL are proto-oncogenes, and their aberrant expression in various cancers has been reported to contribute to cancer metastasis, growth and drug resistance. Aurora kinase B is an effective anticancer drug target. The mechanism of action of G-749 is the inhibition of FLT3 kinase phosphorylation, thereby inhibiting the proliferation of AML and inducing apoptosis (Lee et al., 2014). AXL has been reported to contribute to cancer metastasis, suppression of immune response, and drug resistance in various cancers such as non-small cell lung cancer (NSCLC) and triple-negative breast cancer (TNBC). Inhibition of AXL is known to restore the immune response and, suppress metastasis and drug resistance. G-749 and its derivative SKI-G-801 are potent inhibitors of AXL and exhibit strong inhibition of AXL at nanomolar level (Lee et al., 2014; Vouri and Hafizi, 2017; Wium et al., 2018; Yokoyama et al., 2019; Chen and Liu, 2021). FLT3 kinase, which is mainly expressed in the cerebellum, bone marrow, lymph nodes, thymus, and spleen, is known to be specifically overexpressed in leukemia (Wu et al., 2018b; Cheng et al., 2018). Some FLT3 kinase inhibitors such as MRX-2843, UNC 2025, Giltertinib, and ONO-7475 are dual kinase inhibitors that inhibit TAM (TYRO, AXL, MER) receptor tyrosine kinase (RTK) in non-small cell lung cancer (NSCLC) and breast cancer (Zhang et al., 2014; Minson et al., 2016; Mori et al., 2017; Wu et al., 2018b; Okura et al., 2020). MRX-2843 was first developed as a MER/FLT3 dual kinase inhibitor and exhibited MER-dependent anti-leukemia activity in the FLT3-ITD model. In addition, it has been reported that MRX-2843 can overcome resistance to EGFR inhibitors by inhibiting MER in NSCLC (Minson et al., 2016; Yan et al., 2018). Similar to MRX-2843, UNC2025 was reported to be an MER/FLT3 dual kinase inhibitor (Zhang et al., 2014). Giltertinib and ONO-7475 are AXL/FLT3 dual kinase inhibitors that have been reported to inhibit adaptive resistance to early EGFR-TKI treatment in EGFR-mutant NSCLC (Mori et al., 2017; Okura et al., 2020). Although many FLT3 kinase inhibitors act as dual kinase inhibitors that inhibit TAM RTK, the potential of G-749 as a TAM RTK inhibitor remains unclear.

TYRO3, also known as Tif, Sky, BYK, and Dtk, is a member of the TAM RTK family and is broadly expressed in a variety of cells and tissues (Lan et al., 2000; Lee, 2015). TAM RTKs are activated by ligands, such as protein S and growth arrest-specific protein 6 (Gas6). Protein S is a specific TYRO3 and MER ligand, whereas Gas6 is known to activate all TAM receptors (Hafizi and Dahlback, 2006; Vouri and Hafizi, 2017; Wu et al., 2017; Al Kafri and Hafizi, 2019; Hsu et al., 2019; Al Kafri and Hafizi, 2020). Notably, TYRO3 downstream signaling plays a role in cell survival, proliferation, invasion, metastasis, and apoptosis (Avilla et al., 2011; Kim et al., 2015; Lee, 2015; Chien et al., 2016; Wu et al., 2018a; Smart et al., 2018; Al Kafri and Hafizi, 2019; Fernandez et al., 2019). Activated TYRO3 induces the activation of signaling pathways, including phosphoinositide 3-kinase (PI3K)/AKT, signal transducer and activator of transcription 3 (STAT3), and mitogen-activated protein kinase (MAPK), ultimately promoting cell survival, proliferation, and migration (Lan et al., 2000; Chien et al., 2016; Al Kafri and Hafizi, 2019; Dufour et al., 2019; Aehnlich et al., 2021). Recently, TYRO3 was reported to be overexpressed in various cancers with poor prognoses, such as leukemia, melanoma, thyroid cancer, breast cancer, pancreatic cancer, and ovarian cancer (Lee-Sherick et al., 2013; Kim et al., 2015; Lee, 2015; Smart et al., 2018; Fernandez et al., 2019; Morimoto et al., 2020; Chen and Liu, 2021; Post et al., 2021). According to a previous report, TYRO3 and AXL were not expressed in normal thyroid cells, but showed remarkably increased expression in thyroid cancer cells, thereby contributing to the resistance of thyroid cancer to existing targeted treatments (Avilla et al., 2011). In addition, TYRO3 overexpression in colorectal cancer (CRC) patients is correlated with patient survival. TYRO3 is overexpressed in polyp and colon cancers, but is rarely expressed in normal tissues. Furthermore, in a recent study using TYRO3 antibodies for the treatment of colon cancer, TYRO3 inhibited epithelial-mesenchymal transition (EMT) in colon cancer cells (Chien et al., 2016). Taken together, these studies suggest that overexpressed TYRO3 is associated with tumorigenesis, cancer progression, and metastasis, and is an effective drug target for cancer treatment.

Regulation of abnormally expressed or activated RTKs is a target for tumor growth inhibition (Lee-Sherick et al., 2013; Graham et al., 2014). In tumorigenesis due to aberrant activation or expression of TAM RTKs, we hypothesized that G-749 might contribute to the inhibition of tumorigenesis by inhibiting TAM RTK. Identification of the effect of G-749 on TAM RTK will help explain tumorigenesis caused by TAM RTKs and shed light on the underlying mechanisms for developing novel drugs for cancer treatment. In this study, we investigated whether it is possible to inhibit the activity or expression of TAM RTK by G-749 *in vitro* and *in vivo*, followed by signaling pathways involved in cell proliferation and apoptosis. In our study, G-749 significantly reduced the viability of colon cancer cell lines in a concentration-dependent manner and promoted the degradation of TYRO3 protein through the regulated intramembrane proteolysis (RIP) process. In addition, G-749 reduced STAT3 and AKT phosphorylation in HCT15 and SW620 colon cancer cells and promoted apoptosis. Similar to the *in vitro* results, TYRO3 protein was significantly reduced by G-749 in a xenograft mouse model, and phosphorylation levels of STAT3 and AKT were decreased. Consequently, we propose the potential of G-749 as a novel anticancer drug targeting TYRO3 in colon cancer.

## Materials and Methods

### Reagents and Antibodies

G-749, MG132, z-VAD-FMK, GM6001 and Necrostatin-1 were purchased from Selleckchem (Houston, TX, United States). Hydroxypropyl-β-cyclodextrin, cycloheximide, crystal violet and chloroquine were purchased from Sigma Chemical Co. (St. Louis, MO, United States). Compound E was purchased from MedChemExpress (Monmouth Junction, NJ, United States). The antibodies used in this study are shown in Supplementary Table S1.

### Cell Culture

The THP1, DLD1, HCT15, SW480, SW620, LoVo, and HT29 cell lines were obtained from the Korean Cell Line Bank (KCLB, Seoul, Korea). CCD-18co, human normal colon fibroblast cells were kindly provided by Dr. Mi-Young Son (KRIBB, Daejeon, South Korea). The human monocytic cell line THP-1 and colon cancer cell lines DLD1, HCT15, SW480, SW620, LoVo, CCD-18co and HT29 were cultured in RPMI-1640 medium supplemented with 10% fetal bovine serum (FBS) and 1% antibiotic-antimycotic solution. All cells were maintained at 37°C in a humidified atmosphere containing 5% CO_2_.

### siRNA Transfection

HCT15 and SW620 cells were seeded in 6-well plates and incubated at 37°C in a humidified atmosphere containing 5% CO_2_ for 24 h. After 24 h, Lipofectamine RNAiMAX transfection reagent (Invitrogen; Thermo Fisher Scientific, Inc., Waltham, MA, United States) was used for transient transfection of cells with Negative control siRNA, TYRO3 siRNA, AXL siRNA and MER siRNA (30 nM, respectively) for 72 h, following the manufacturer’s protocol. The siRNA sequences are listed in Supplementary Table S2.

### Cell Viability Assay

Cell viability was measured using an MTS assay kit (Promega, Madison, WI, United States). The cells were seeded in 96-well plates and incubated at 37°C in a humidified atmosphere containing 5% CO_2_ for 24 h (cancer cell lines) or 48 h (CCD-18co cell line) and then treated with various concentrations of G-749 diluted in fresh RPMI-1640 medium. After 24, 48, and 72 h, the MTS solution was added to each well for 2 h before measuring the absorbance at 490 nm with a microplate reader (Spectramax i3X, Molecular Devices, San Jose, CA, United States).

### Crystal Violet Staining

HCT15, SW620, and CCD-18co cells were seeded in 12-well plates and incubated at 37°C in a humidified atmosphere containing 5% CO_2_ for 24 h (HCT15 and SW620) or 48 h (CCD-18co) before treatment with G-749. The cells were treated with G-749 at the indicated concentrations for 48 h and then fixed with 4% paraformaldehyde (PFA) for 15 min at room temperature. The fixed cells were stained with 0.5% crystal violet solution for 15 min. Images were visualized using an Epson Perfection V700 Photo scanner.

### RNA Isolation and RT-PCR Analysis

Total RNA was extracted using TRIzol reagent (Thermo Fisher Scientific) according to the manufacturer’s instructions. First-strand cDNA was synthesized from 1 μg of total RNA using a reverse transcription PCR kit (Enzynomics, Daejeon, Korea). After cDNA synthesis, conventional PCR was performed using cDNA as a template. The primer sequences are described in Supplementary Table S3.

### Flow Cytometry Analysis

For the measurement of the Sub G1 content, the cells were suspended in 200 μl of phosphate-buffered saline (PBS) containing 50% FBS, after which 700 μl of 95% ethanol was added while the cells were vortexed. The cells were then incubated at −20°C overnight, washed with PBS, resuspended in 200 μl of 1.12% sodium citrate buffer (pH 8.4) with 12.5 μg RNase, and incubated for 15 min at room temperature. DNA was stained with propidium iodide solution (50 μg/ml) for 15 min at room temperature. DNA content was quantified using a FACS machine (Attune NxT, Thermo Fisher Scientific). The FITC Annexin V apoptosis detection kit was used to determine cell death according to the manufacturer’s instructions (BD Bioscience, San Jose, CA, United States). To measure TYRO3 expression on the cell surface, cells were fixed with 2% PFA for 15 min at room temperature and then permeabilized with 0.1% digitonin. After permeabilization, the cells were stained with PE-conjugated TYRO3 (N-terminal) or PE-conjugated IgG control antibodies for 30 min at 4°C.

### Western Blot Analysis

For western blot analysis, the cells were washed with cold PBS and lysed with RIPA lysis buffer containing 1X protease and 1X phosphatase inhibitor cocktail (Roche, Diagnostics GmbH, Mannheim, Germany). The lysates were centrifuged at 13,000 rpm for 30 min at 4°C. The amount of protein was quantified using the Bradford assay. Equal amounts of protein were separated by SDS-PAGE and transferred onto polyvinylidene fluoride membranes. The membranes were then blocked with 5% skim milk in Tris-buffered saline containing 0.1% Tween-20 (TBST) for 1 h at room temperature and then incubated with primary antibodies in 3% BSA diluted in TBST for 4 h at room temperature. Afterwards, the membranes were incubated with HRP-conjugated secondary antibodies for 1 h at room temperature, and the bands were detected using an enhanced chemiluminescence (ECL) solution and visualized using Lumino-Graph I (ATTO, Tokyo, Japan).

### *In vivo* Tumor Xenograft Model

BALB/c male nude mice (18–20 g, 5 weeks of age) were purchased from Orient Bio Inc. (Seongnam, Korea) and housed in a facility at 55–60% humidity and controlled temperature of 24 ± 3°C with a light/dark cycle of 12 h. The mice were provided with food and water *ad libitum*. HCT15 cancer cells (1 × 10^7^ cells/mouse) were subcutaneously injected into the nude mice at 6 weeks of age. When the average tumor volume reached approximately 100 mm^3^, the mice were randomized into vehicle control (20% hydroxypropyl-β-cyclodextrin, HPBCD in water) and treatment groups (*n* = 5). G-749 was suspended in vehicle solution (5, 15, or 30 mpk in 20% hydroxypropyl-β-cyclodextrin, respectively) and administered orally once a day for 18 days. Tumor size was measured every 2 days using a Vernier caliper, and tumor volume (mm^3^) was determined using the following equation: V = length × width^2^/2. Body weight was measured at the same time as the tumor volume.

### TUNEL Assay

For TUNEL staining, 10 μM thick cryosectioned tissues were fixed with 4% PFA and permeabilized by 200 ng/μl Proteinase K solution for 30 min at 37°C. Terminal deoxynucleotide transferase-mediated deoxy-UTP nick end labeling of nuclei was performed using the DeadEnd™ Fluorometric TUNEL System (Promega), according to the manufacturer’s instructions. Nuclei were stained with DAPI. All slides were mounted with Vectashield mounting medium (Vector Laboratories, Paris, France). Images were obtained using fluorescence microscopy.

### Ki-67 Staining

To measure the Ki-67 positive level in cancer tissues, 10 μM thick cryosectioned tissues were fixed with 4% PFA for 15 min at room temperature. Fixed tissues were permeabilized with PBS containing 0.2% Tween-20. The slides were incubated with 4% BSA in PBST (1x PBS with 0.05% Tween-20) for 1 h and then were incubated with a rabbit anti Ki-67 antibody (1:100) overnight at 4°C and incubated with goat anti-Rabbit 488 secondary antibody (1:300) for 1 h at room temperature. The nuclei were stained with DAPI and images were captured using fluorescence microscopy.

### Statistical Analysis

All data are presented as mean ± standard error of the mean (SEM) and were analyzed via one-way analysis of variance (ANOVA) or two-way ANOVA using the GraphPad Prism 5.01 software. All experiments were repeated at least three times. To evaluate statistical significance, one-way ANOVA followed by Tukey’s multiple comparison test and two-way ANOVA followed by Bonferroni post-test were used to compare multiple groups. Statistical significance was set at *p* < 0.05.

## Results

### G-749 Decreases the Viability of Colon Cancer Cells

**FIGURE 1:**
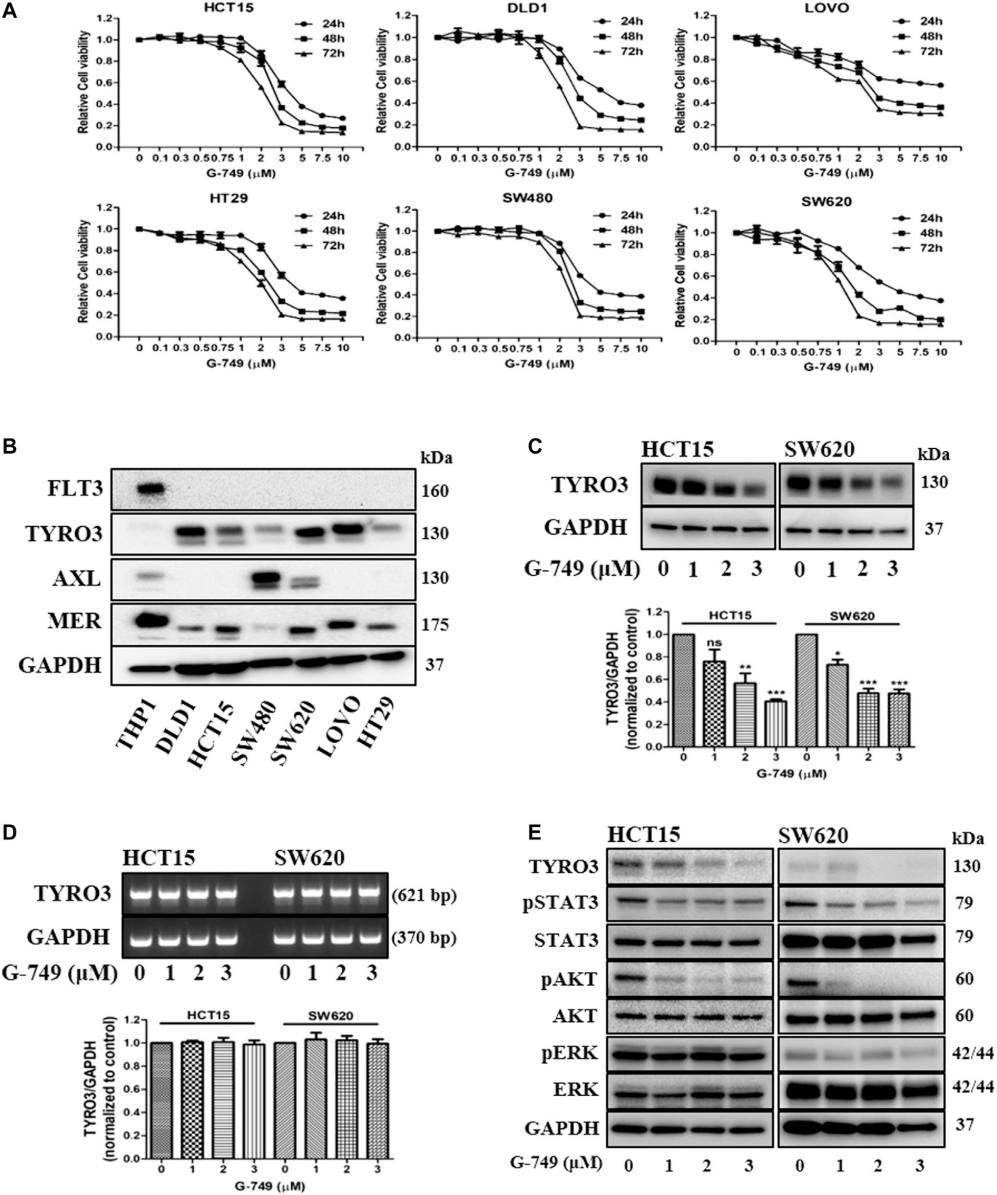
Anticancer effects of G-749 in colon cancer cell lines. **(A)** The viabilities of the colon cancer cell lines were measured using an MTS proliferation assay. Cells were treated with G-749 at the indicated concentrations for 24, 48, and 72 h. **(B)** Expression of TAM receptors and FLT3 kinase was analyzed via western blot using anti-TYRO3, -AXL, -MER, -FLT3 and GAPDH antibodies. **(C)** HCT15 and SW620 cells were treated with G-749 (1, 2, or 3 μM) or DMSO for 24 h. Equal amounts of cell lysates were subjected to electrophoresis and analyzed via western blot to detect TYRO3 levels. GAPDH was used as a loading control. **(D)** RNA levels were determined by RT-PCR analysis. HCT15 and SW620 cells were treated with G-749 (1, 2, or 3 μM) or DMSO for 24 h. GAPDH was used as a control. **(E)** Cells were treated with G-749 at the indicated concentrations for 24 h. Equal amounts of cell lysates were then subjected to electrophoresis and analyzed via western blot using anti-TYRO3, -phospho-STAT3, -STAT3, -phospho-AKT, -AKT, -phospho-ERK, -ERK, and -GAPDH antibodies. GAPDH was used as a loading control. The bar graph was generated by quantifying blots from three independent experiments using ImageJ and normalizing the intensity of the bands to the control. Data are presented as means ± standard deviations (SD). Data were analyzed using one-way ANOVA with Tukey’s multiple comparison post-hoc analysis; ****p < 0.0001, ***p < 0.001, **p < 0.01, *p < 0.05.

To verify whether G-749 exhibits anticancer effects in colon cancer cells, we first investigated cell viability in six colon cancer cell lines, HCT15, DLD1, LoVo, HT29, SW480 and SW620. Treatment of cells with G-749 at varying concentrations for 24, 48, and 72 h, was effective at reducing viability in the micromolar range in a dose-dependent manner (Figure 1A). In addition, G-749 effectively reduced the viability in the micromolar range in a variety of human cancer cells (Supplementary Figure S1). Furthermore, we investigated the toxicity of G-749 in human normal fibroblast cells, CCD-18co via crystal violet staining and an MTS assay. Results showed that G-749 was less toxic to CCD-18co normal colon fibroblast cells compared to colon cancer cells (Supplementary Figures S2A,B). Next, we investigated the expression of FLT3 kinase and TAM RTKs in colon cancer cells using western blot analysis. MER and TYRO3 were expressed in all the colon cancer cell lines. However, FLT3 kinase was not expressed in any of the colon cancer cell lines, but was expressed in the THP-1 leukemia cell lines. Furthermore, AXL was expressed in only two colon cancer cell lines, SW480 and SW620, and was not expressed in DLD1, HCT15, LoVo, and HT29 cells (Figure 1B). Interestingly, we found that TYRO3 protein levels decreased in a concentration-dependent manner when colon cancer cells were treated with G-749 (Figure 1C). Thus, we chose to further investigate the molecular mechanism by which G-749 reduced TYRO3 protein levels and assessed TYRO3 mRNA levels to determine whether protein translation or degradation was involved. We found that treatment with G-749 at various concentrations for 24 h did not alter mRNA levels, but instead led to a decrease in protein levels (Figures 1C,D). To investigate the effect of G-749 on cell proliferation, we analyzed the signaling pathways involved in cell proliferation by western blot analysis. When cells were treated with G-749, TYRO3 expression was markedly reduced. In addition, the phosphorylation of STAT3 and AKT was significantly decreased in a concentration-dependent manner, whereas phosphorylation of ERK was not altered. These results suggest that G-749 regulates the phosphorylation of proteins such as STAT3 and AKT, thereby regulating the signaling pathways involved in cell proliferation (Figure 1E). Taken together, we hypothesized that G-749 effectively reduced phosphorylation of STAT3 and AKT, which are involved in cell proliferation, through the reduction of TYRO3 in colon cancer cells.

### Knock-Down of TYRO3 Reduces Cell Viability and Induces Apoptosis in Colon Cancer Cells

**FIGURE 2:**
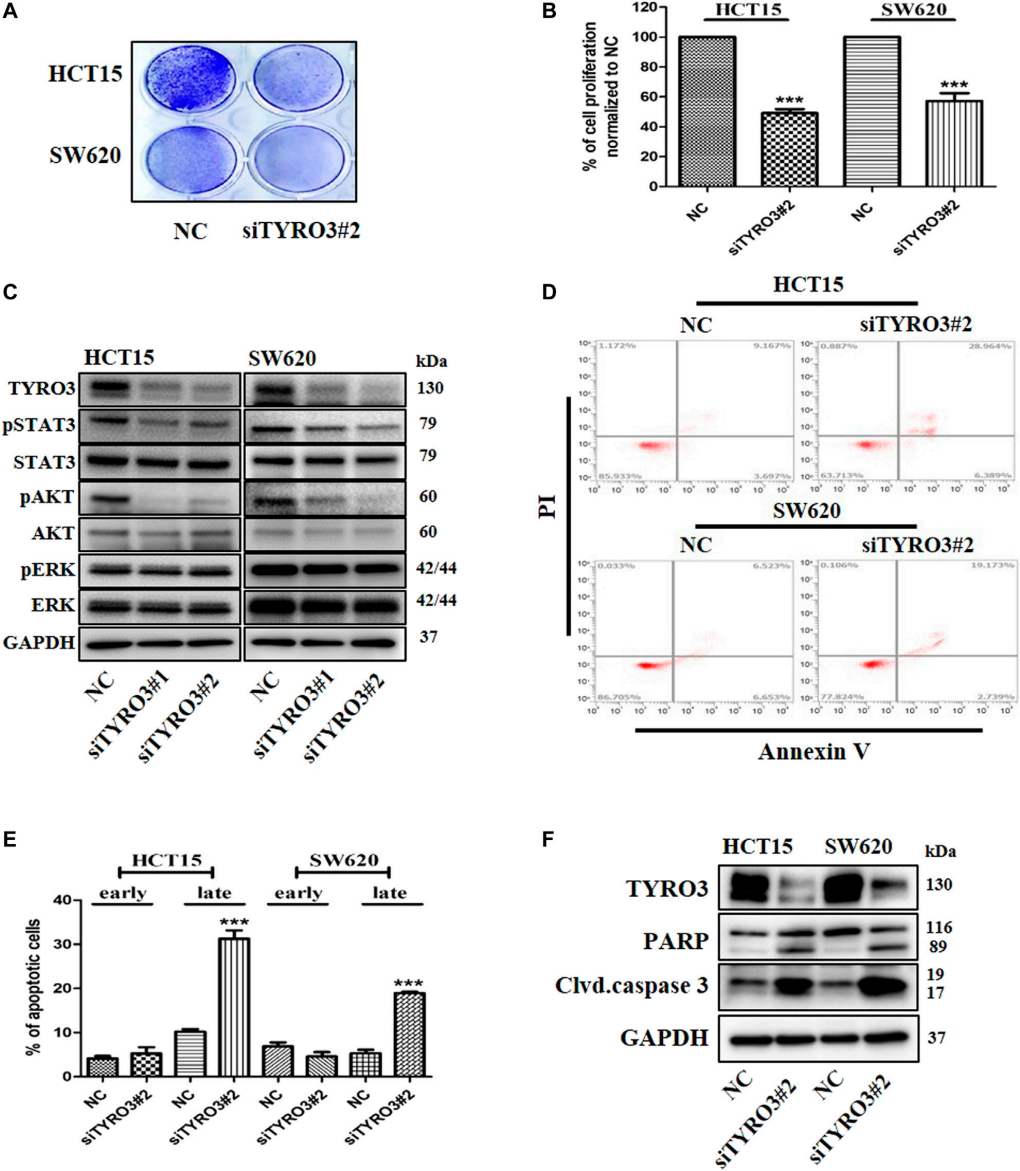
Knock-down of TYRO3 by siRNA inhibits cell proliferation and induces apoptosis in colon cancer cell lines. **(A)** Cells were transfected with TYRO3, and negative control (NC) siRNA for 72 h. Cells were stained with 0.5% crystal violet. **(B)** Cells were incubated for 72 h after transfection with TYRO3, and NC siRNA. The viabilities of the transfected cells were measured using an MTS proliferation assay. **(C)** HCT15 and SW620 cells were transfected with TYRO3, and NC siRNA for 72 h. Equal amounts of cell lysates were then subjected to electrophoresis and analyzed via western blot using anti-TYRO3, -phospho-STAT3, -STAT3, -phospho-AKT, -AKT, -phospho-ERK, -ERK, and–GAPDH antibodies. **(D,E)** HCT15 and SW620 cells were transfected with siRNA for 72 h. Apoptotic cells were stained with propidium iodide/Annexin V and then analyzed by flow cytometry. Early apoptotic cells and late apoptotic cells are displayed as bar graph. **(F)** Cells were transfected with TYRO3, and NC siRNA for 72 h and then analyzed via western blot using anti-PARP, -cleaved caspase 3 and–GAPDH antibodies. Data are presented as means ± standard deviations (SD). Data were analyzed using one-way ANOVA with Tukey’s multiple comparison post-hoc analysis; ****p < 0.0001, ***p < 0.001, **p < 0.01, *p < 0.05.

To further investigate whether TAM RTKs can induce cell proliferation and apoptosis in colon cancer cells, we downregulated the expression of TAM RTKs using specific siRNAs targeting TYRO3, AXL and MER. When crystal violet staining was performed after down-regulating TAM RTKs in colon cancer cells, we found that the number of cells in the TYRO3-downregulated group was significantly reduced compared to the MER- and AXL-downregulated groups. In addition, we found that the number of cells in the MER-downregulated group was slightly reduced compared to that in NC group. In contrast, there was no decrease in the number of cells in the AXL-downregulated group compared to the NC group (Figure 2A and Supplementary Figure S3A). Furthermore, we measured cell viability using an MTS assay. Similar to crystal violet staining, we found that the TYRO3-downregulated group had significantly reduced cell viability compared to the control group. In addition, cell viability was slightly decreased in the MER-downregulated group compared to the control group, whereas the AXL-downregulated group showed no change in cell viability (Figure 2B and Supplementary Figure S3B). To investigate the effect of siRNA targeting TYRO3, AXL and MER on signaling pathways, we analyzed the signaling pathways involved in cell proliferation by western blot analysis in colon cancer cells. We found that phosphorylation of STAT3 and AKT was decreased when TYRO3 and MER were silenced in HCT15 and SW620 cells but not AXL (Figure 2C and Supplementary Figure S3C). We further investigated whether silencing of TAM RTKs in colon cancer cells could induce apoptosis. As a result, apoptosis was significantly increased in the TYRO3 silencing group compared to the NC siRNA group. In the group with silenced MER, apoptosis was partially increased compared to that in the TYRO3 group. In contrast, apoptosis in the AXL-silenced group was not different from that in the NC group (Figures 2D,E, Supplementary Figure S3D). To elucidate the more precise molecular mechanism, we investigated the molecules involved in apoptosis by western blot analysis. When colon cancer cells were transfected with siRNA targeting TYRO3, AXL, MER, or NC, it was found that the cleaved form of poly (ADP-ribose) polymerase (PARP) and caspase 3 were significantly increased (Figure 2F and Supplementary Figure S3E). Taken together, these results suggest that TYRO3 and MER may be drug targets for G-749 in colon cancer.

### G-749 Accelerates Proteasomal Degradation Through Presenilin-Dependent Cleavage of TYRO3 in Colon Cancer Cells

**FIGURE 3:**
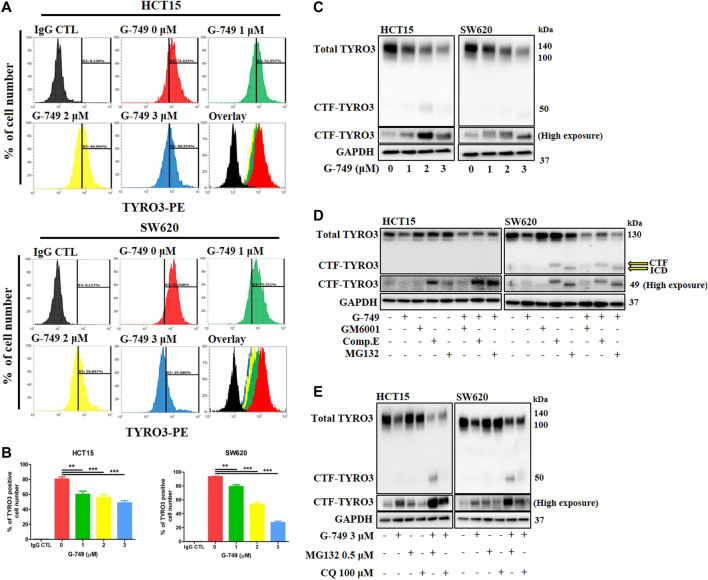
G-749 promotes PS-RIP-dependent TYRO3 cleavage and proteasomal degradation in colon cancer cell lines. **(A,B)** Cells were treated with G-749 (1, 2 or 3 μM) or DMSO for 24 h and then stained with PE-conjugated TYRO3 (N-terminal) and PE-conjugated IgG control antibodies. Surface expression of TYRO3 was analyzed by flow cytometry. **(C)** HCT15 and SW620 cells were treated with G-749 for 12 h. Total TYRO3 and C-terminal fragment of TYRO3 levels were detected using an antibody against the C-terminal region of TYRO3. GAPDH was used as a loading control. **(D)** Cells were pre-treated with GM6001 (30 μM) for 12 h and then additionally treated with G-749 (3 μM), Compound E (1 μM) and MG132 (0.5 μM) for 12 h. The incubated cells were lysed and analyzed by western blot with anti-TYRO3 and–GAPDH antibodies. **(E)** Cells were co-treated with G-749 (3 μM) and either MG132 (0.5 μM) or CQ (100 μM) for 12 h. Total TYRO3 and C-terminal fragment of TYRO3 levels were detected using an antibody against the C-terminal region of TYRO3. Data are presented as means ± standard deviations (SD). Data were analyzed using one-way ANOVA with Tukey’s multiple comparison post-hoc analysis; ****p < 0.0001, ***p < 0.001, **p < 0.01, *p < 0.05.

We hypothesized that the G-749-induced decrease in TYRO3 levels may be associated with protein stability and thereby performed a cycloheximide (CHX) chase assay. When cells were co-treated with G-749 and the protein synthesis inhibitor CHX, TYRO3 was markedly reduced compared to cells treated with G-749 or CHX alone (Supplementary Figure S4A). We further evaluated whether the G-749-induced reduction in TYRO3 expression levels was reversible or irreversible. After 8 h of treatment with G-749, cells were either washed or unwashed. TYRO3 protein expression levels were remarkably decreased in the unwashed group, whereas they were recovered in the washed group (Supplementary Figure S4B). These results indicate that the decrease in TYRO3 expression induced by G-749 is reversible. In the present study, we found that the RNA expression of TYRO3 was not altered by treatment with G-749. In addition, many RTKs have been reported to be regulated by RIP (Merilahti et al., 2017). Therefore, we tested whether the G-749-induced decrease in TYRO3 levels was associated with its degradation via RIP. We first investigated the decrease in the membrane expression of TYRO3 via flow cytometry. The expression of TYRO3 was analyzed using TYRO3 phycoerythrin (PE) -conjugated antibody (N-terminal) or control IgG PE-conjugated antibody. Similar to the western blot analysis results, when cells were treated with various concentrations of G-749, TYRO3 expression in the cell membrane was significantly reduced in a concentration-dependent manner compared to the control group (Figures 3A,B). Next, we further investigated whether the C-terminal fragment (CTF) of TYRO3 accumulates in the cytoplasm through western blot analysis. When colon cancer cell lines were treated with G-749 at various concentrations for 12 h, the total TYRO3 (110, 130 kDa) protein levels were significantly decreased, whereas TYRO3 CTF (49 kDa) levels were markedly increased in a concentration-dependent manner (Figure 3C). To determine whether the increased CTF production of TYRO3 by G-749 is dependent on the RIP process, we used GM6001, a pan-MMP inhibitor, and Compound E, a gamma-secretase complex inhibitor. In the group treated with G-749 alone, it was confirmed that the generation of CTF increased at 49 kDa; in the group co-treated with GM6001 and G-749, the generation of CTF was not inhibited. The gamma-secretase cleaves proteins anchored to the cell membrane and releases the intracellular domain (ICD) into the cytoplasm (Brown et al., 2000; Lu et al., 2017). Similarly, the cell membrane-anchored CTF of TYRO3 produced by G-749 is cleaved into ICD by the gamma-secretase complex and is relased into the cytoplasm. This process was completely blocked when the cells were co-treated with G-749 and Compound E (Figure 3D). In addition, the expressions of ADAM10 (A Disintegrin and Metalloproteinase domain-containing protein 10), ADAM17 (A Disintegrin and Metalloproteinase 17, also called TACE), PSEN1 (presenilin-1) and PSEN2 (presenilin-2), which are key proteins in the RIP process, were not altered by G-749 treatment (Supplementary Figures S5A,B). To further test whether proteasomal or lysosomal degradation is associated with the G-749-induced decrease in TYRO3 levels, cells were treated with G-749 and either the proteasomal inhibitor MG132 or the lysosomal inhibitor chloroquine (CQ). We identified an approximately 49 kDa TYRO3 CTF in cells treated with G-749 and MG132 (Figures 3D,E), suggesting that proteasome-dependent degradation is involved in this process. In summary, these results suggest that TYRO3 degradation by G-749 is mediated by presenilin-dependent cleavage but not by MMPs.

### G-749 Induces Caspase-Dependent Apoptosis in Colon Cancer Cells

**FIGURE 4:**
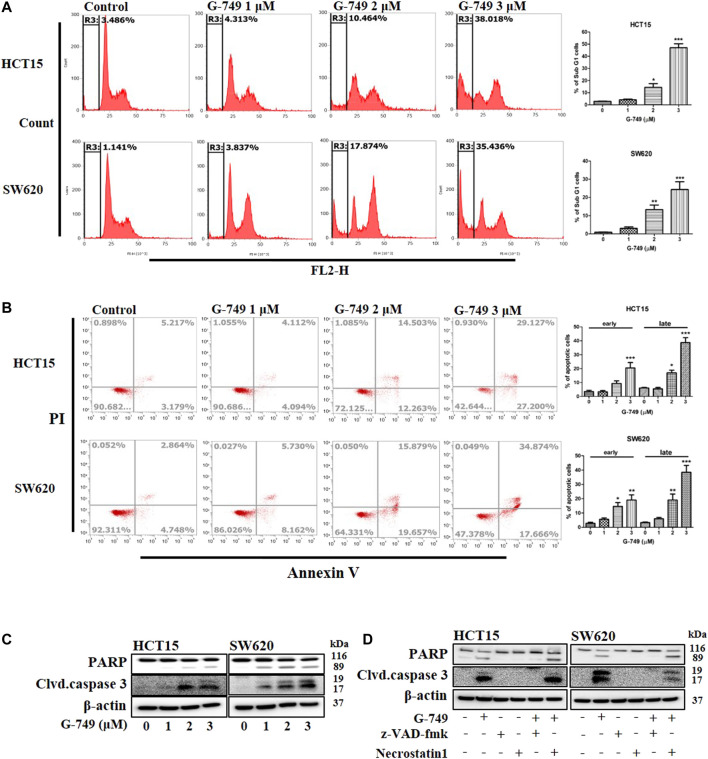
G-749 induces apoptosis in colon cancer cell lines. **(A)** HCT15 and SW620 cells were treated with the indicated concentration of G-749 for 36 h and then stained with propidium iodide. The SubG1 population was analyzed by flow cytometry. **(B)** Cells were treated with the indicated concentration of G-749 for 36 h. Apoptotic cells were stained with propidium iodide/Annexin V and then analyzed by flow cytometry. **(C)** Cells were treated with G-749 for 36 h. The cell lysates were subjected to electrophoresis and analyzed by western blot with antibodies against PARP and cleaved caspase 3. β-actin was used as a loading control. **(D)** HCT15 and SW620 cells were co-incubated with G-749 (3 μM), z-VAD-fmk (50 μM), and Necrostatin 1 (30 μM) for 36 h and analyzed by western blot with antibodies against PARP, cleaved caspase 3 and β-actin. Data are presented as means ± standard deviation (SD). Data were analyzed via one-way ANOVA with Tukey’s multiple comparison post-hoc analysis; ****p < 0.0001, ***p < 0.001, **p < 0.01, *p < 0.05.

To determine whether G-749 could induce apoptosis in colon cancer cells, we investigated apoptosis using flow cytometry. We found that when cells were treated with various concentrations of G-749, the SubG1 cell population of cancer cells increased proportionally to the concentration of G-749 used (Figure 4A). In addition, Annexin V staining confirmed that apoptosis increased after G-749 treatment in a concentration-dependent manner (Figure 4B). To identify the more precise molecular mechanism, we investigated the molecules involved in apoptosis by western blot analysis. When colon cancer cells were treated with various concentrations of G-749, the cleaved form of PARP was significantly increased. In addition, the cleaved form of caspase 3 increased in a concentration-dependent manner (Figure 4C). The cleavage of PARP and caspase 3 was completely inhibited by co-treatment with the pan-caspase inhibitor, z-VAD-FMK. In contrast, treatment with necrostatin 1 did not inhibit G-749-induced cleavage of PARP and caspase 3 (Figure 4D). Taken together, these data suggest that apoptosis is an appropriate mechanism of cell death induced by G-749 treatment.

### G-749 Suppresses Tumor Growth in Mouse Xenograft Models

**FIGURE 5:**
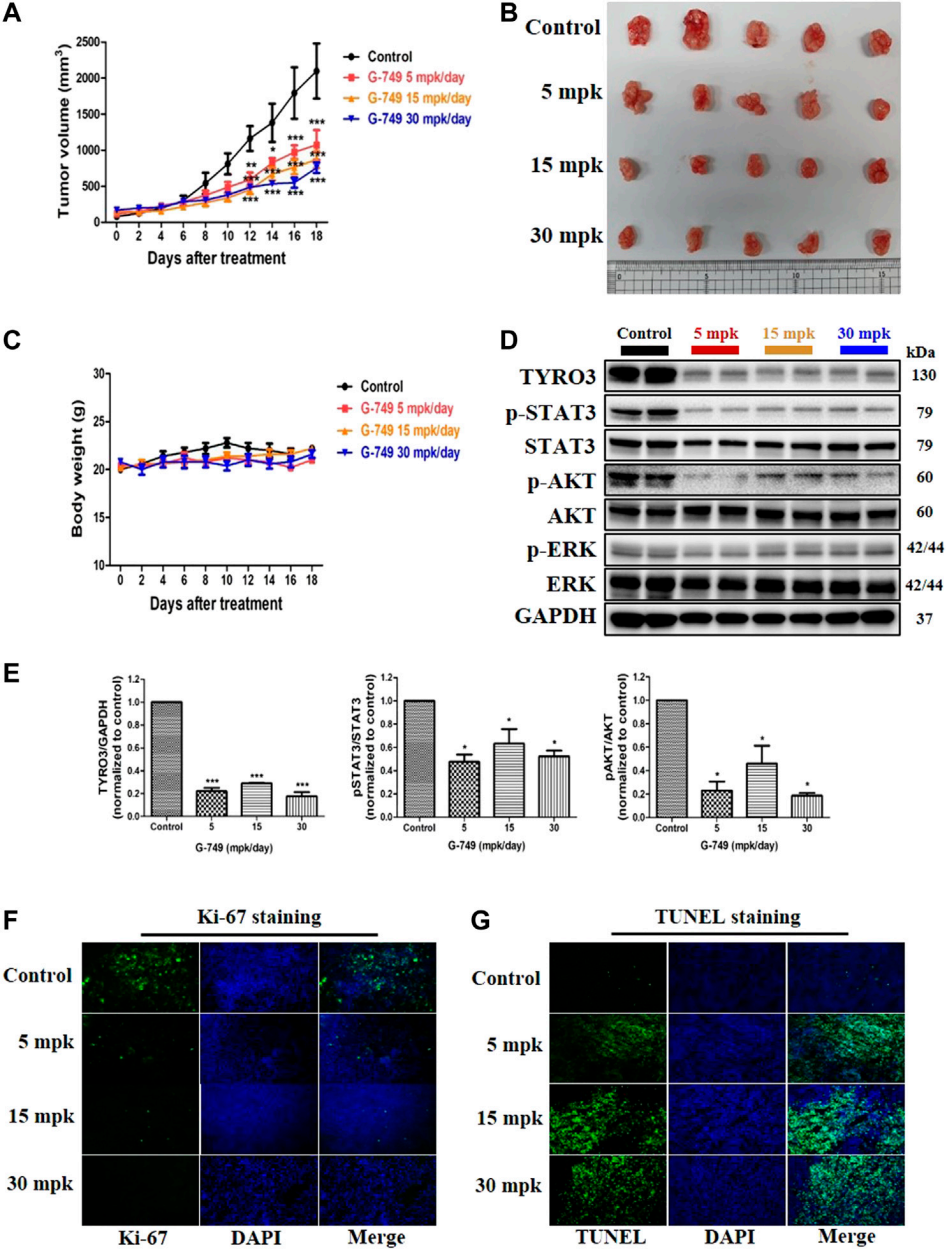
G-749 suppresses tumor growth and TYRO3 expression in a tumor xenograft mouse model. **(A)** HCT15 cells were inoculated subcutaneously into the flanks of BALB/c-nude mice. When the tumor size reached approximately 100 mm^3^ in volume, HCT15 tumor-bearing mice received oral administration of Vehicle (20% β-cyclodextrin) or G-749 (5, 15, and 30 mpk/day in 20% β-cyclodextrin) each day for 18 days. The tumor volumes were measured every 2 days (*n* = 5 mice per group). **(B)** Representative photographs of the excised HCT15 tumors from four groups. **(C)** The body weight change was monitored in the xenograft model throughout the treatment. **(D,E)** Xenograft tumor tissues in each treatment group were analyzed by western blotting. **(F)** Immunofluorescence was performed on 10 μM thick sectioned tumor tissues to detect Ki-67 protein. **(G)** Representative images of the TUNEL assay on G-749 treated tumor tissues. The bar graph was generated by quantifying blots using ImageJ and normalizing the intensity of the bands to control. Data are presented as means ± standard deviation (SD). Data are analyzed via one-way ANOVA with Tukey’s multiple comparison post-hoc analysis or two-way ANOVA with Bonferroni post analysis; ****p < 0.0001, ***p < 0.001, **p < 0.01, *p < 0.05.

We further evaluated the antitumor effects of G-749 in BALB/c nude mouse tumor xenograft models inoculated with HCT15 cells. BALB/c-nude mice received orally administered G-749 once daily at 5, 15, or 30 mg of base/kg of body weight (mpk) suspended in 20% β-cyclodextrin. The control group mice received 20% β-cyclodextrin orally. Similar to the *in vitro* results, G-749 treatment significantly suppressed tumor growth in xenograft mice (Figures 5A,B). Moreover, there was no significant difference in body weight between the drug treatment and control groups (Figure 5C). To confirm the antitumor effect of G-749 in xenograft mouse models, the total TYRO3, phosphorylated-STAT3, -AKT, and -ERK were compared by western blotting. In agreement with previous *in vitro* results, TYRO3 protein levels were significantly reduced by G-749 *in vivo*. Moreover, the phosphorylation of STAT3 and AKT was significantly reduced in the groups treated with G-749 at 5, 15, and 30 mpk, when compared to the control group (Figures 5D,E). In addition, we performed Ki-67 staining and TUNEL assay and found that the number of Ki-67 positive staining was decreased, whereas TUNEL staining was increased by G-749 in a dose-dependent manner (Figures 5F,G). Thus, these results indicate that G-749 effectively suppresses tumor growth by inhibiting the phosphorylation of STAT3 and AKT via a decrease in TYRO3 levels in a xenograft mouse model.

## Discussion

According to recent studies, the TAM RTK family plays an important role in various cancers (Post et al., 2021). Among them, AXL and MER are known proto-oncogenes and have been confirmed to be overexpressed in several cancer types (Rothlin et al., 2014; Morimoto et al., 2020; Ruicci et al., 2020). While AXL and MER have been thoroughly investigated, the role of TYRO3 in cancer development remains unknown. However, a growing number of reports have shown that TYRO3 is overexpressed in various cancers with poor prognoses, and it has been proposed as a potential target for cancer treatment (Smart et al., 2018; Hsu et al., 2019). A number of studies have shown that FLT3 kinase inhibitors are dual kinase inhibitors that inhibit the TAM RTK family (Zhang et al., 2014; Minson et al., 2016; Mori et al., 2017; Yan et al., 2018; Okura et al., 2020). For example, MRX-2843 abrogates the downstream signaling effectors of the TAM RTK family by inhibiting FLT3 and MER. In addition, MRX-2843 inhibits colony formation and induces apoptosis in AML cells (Minson et al., 2016). Furthermore, the anticancer effects of MRX-2843 have been shown to be effective in overcoming resistance to EGFR-TKIs in NSCLC (Yan et al., 2018). Other examples include Giltertinib and ONO-7475, which are dual kinase inhibitors that inhibit AXL and FLT3 kinase (Mori et al., 2017; Okura et al., 2020). Therefore, FLT3 kinase inhibitors are thought to be correlated with the TAM RTK family. G-749, designated as an orphan drug by the FDA, inhibits the phosphorylation of FLT3 kinase and proliferation, and also induces apoptosis in acute myeloid leukemia cells. Although G-749 was previously reported to inhibit MER and AXL in a kinase assay, its potential as an anticancer therapy and the detailed mechanism as a TAM RTK inhibitor has not been clearly elucidated (Lee et al., 2014). Based on previous studies, we hypothesized that G-749 could be a novel drug targeting TAM RTKs in solid cancers. We first investigated the anticancer effect of G-749 in lung, liver, ovarian, prostate, stomach, and colon cancer cell lines via an MTS assay and found that it was effective at reducing viability in the micromolar range in a variety of human cancer cell lines. In addition, the toxicity of G-749 in CCD-18co normal colon fibroblast cells was lower than that in colon cancer cells. These results suggest that G-749 may act as an effective anticancer drug in various cancers. Next, we investigated the expression of FLT3 kinase and TAM RTKs in colon cancer cells. MER and TYRO3 were expressed in all the colon cancer cell lines. However, FLT3 kinase was not expressed in any of the colon cancer cell lines, but was expressed in the leukemia cell line THP-1. Furthermore, AXL was expressed in only two colon cancer cell lines, SW480 and SW620, and was not expressed in DLD1, HCT15, LoVo, and HT29 cells. These results suggest that reduced colon cancer cell viability is not dependent on FLT3 kinase. Interestingly, we found that the expression of TYRO3 protein was decreased by G-749 in a concentration-dependent manner but not by the expression of TYRO3 RNA. These results suggest that the reduction of TYRO3 by G-749 is regulated at the protein level.

The activation of STAT3 is associated with cell proliferation and the inhibition of apoptosis in breast and non-small cell lung cancer. Conversely, inhibition of the JAK/STAT3 signaling pathway slows cancer cell growth and induces apoptosis in various cancers (Zhang et al., 2003; Alvarez et al., 2006; Choudhari et al., 2007; Al Zaid Siddiquee and Turkson, 2008). In addition, constitutively activated STAT3 promotes the phosphorylation of AKT, which is involved in the proliferation of various cancers (Al Zaid Siddiquee and Turkson, 2008). In previous reports, inhibition of JAK1 and 2/STAT3 signaling was reported to induce apoptosis and cell cycle arrest in colorectal cancer cells (Xiong et al., 2008). Down-regulation of TYRO3 via RNAi in breast, colon, head and neck and pancreatic cancers has been reported to regulate the cellular signaling pathway by reducing the phosphorylation of STAT3, AKT, and ERK. Conversely, the overexpression of TYRO3 has been shown to increase the phosphorylation of ERK and AKT (Lan et al., 2000; Ekyalongo et al., 2014; Duan et al., 2016; Qin and QIAN, 2018; Morimoto et al., 2020; Ruicci et al., 2020; Tsai et al., 2020). AKT and ERK are key mediators in regulating the survival and proliferation of cancer cells (Herrero et al., 2015; Song et al., 2019), whereas STAT3 is a transcription factor involved in cell survival and apoptosis (Xiong et al., 2008; Lee et al., 2019). Therefore, we investigated the effect of G-749 treatment on the signaling pathways involved in cell proliferation. As a result, phosphorylation of STAT3 and AKT but not ERK was significantly reduced when cells were treated with G-749. To identify the more detailed roles of TAM RTKs and their molecular mechanisms, we used specific siRNA targeting TAM RTKs in colon cancer cells. Knockdown of TYRO3 and MER, but not AXL reduced cell viability and increased apoptosis. We also found that phosphorylation of STAT3 and AKT was decreased in the group with downregulated TYRO3 and MER but not AXL. Furthermore, the cleavage forms of PARP and caspase 3 associated with apoptosis were increased in the group with downregulated TYRO3 and MER. In previous reports, AXL in colon cancer was not correlated with patient survival (Schmitz et al., 2016). It has also been reported that downregulation of AXL in colon cancer cells is involved in metastasis rather than in cell proliferation (Uribe et al., 2017).

Although TAM RTKs share the same signaling pathway, the inhibition of each TAM RTK results in different responses in signaling pathways. In renal cancer, downregulation of AXL reduced phosphorylation of AKT but not phosphorylation of ERK (Yu et al., 2015). In contrast, knockdown of AXL in MDA-MB-231 cells did not affect the AKT signaling pathway and did not induce apoptosis (Sinha et al., 2015). Similar to other TAM RTKs, TYRO3 was also shown to activate common oncogenic signaling pathways, such as MEK/ERK and PI3K/AKT, in tumor cells (Wong et al., 2019). In particular, numerous studies have reported that the PI3K/AKT pathway, a downstream signaling pathway of TYRO3, plays an important role in cancer cells (Tsai et al., 2020). Supporting this claim, activation of the PI3K/AKT signaling pathway in response to stimuli, such as Gas6 and Protein S, was shown to be attenuated in TYRO3 knockout mice. Additionally, TYRO3 overexpression in fibroblast-like Rat2 cells has been shown to increase AKT phosphorylation (Angelillo-Scherrer et al., 2005; Brown et al., 2012; Smart et al., 2018). Furthermore, inhibition of TYRO3 by siRNA or shRNA in hepatocellular carcinoma, CRC, and breast cancer cell lines directly reduce PI3K/AKT phosphorylation (Duan et al., 2016; Tsai et al., 2020). Interestingly, downregulation of TYRO3 in various breast cancer cell lines resulted in cell-specific responses to signaling pathways (Ekyalongo et al., 2014). Downstream signaling pathways following activation of each TAM receptor are well described in EGFR/TAM chimeric models. In this chimeric model, a unique post-receptor function of the TAM receptor was revealed. TAM receptors show distinct arrangements of autophosphorylation sites. In addition, differences in the kinetics and strengths of the activity profiles of signaling pathway molecules, such as phosphorylated ERK, AKT, and RSK2, were reported for each TAM receptor. Furthermore, the gene expression profiles did not overlap for each TAM receptor in the chimeric model (Kimani et al., 2016). In summary, the signaling pathways after each TAM receptor activation may differ, and the downstream signaling of each receptor may be tumor- or cell-specific. In addition, it may vary depending on the cell conditions. In this study, we demonstrated the potential of TYRO3 and MER as drug targets in colon cancer cells. However, our knockdown results revealed that TYRO3 is a more effective drug target than MER in colon cancer cells.

In the early stage of colon cancer, TYRO3 overexpression is associated with cancer development, as its aberrant expression promotes tumorigenesis (Chien et al., 2016). In addition, TYRO3 mutations have been identified in various cancers, and none have been reported to be associated with cancer development. Therefore, inhibition of overexpression or aberrant activity of TYRO3 is an effective drug target for tumorigenesis (Rothlin et al., 2014; Smart et al., 2018; Post et al., 2021). Recently, RTKs have been shown to transmit intracellular signals through a process called RIP, which regulates cellular behavior via a cleaved intracellular domain (ICD) (Ancot et al., 2009; Ancot et al., 2012; Lu et al., 2017). In this study, we found that TYRO3 protein levels are regulated by proteolytic cleavage processes, such as RIP. In the RIP process, MMPs, disintegrins, and ADAMs are involved in cleaving the extracellular domain, also known as ectodomain shedding. Subsequently, membrane-anchored CTF is released into the cytoplasm by the γ-secretase complex. The ICD produced by the RIP process is degraded by the proteasome pathway, while some are used to transmit cellular signals. In our study, CHX chase assay revealed that G-749 reduced the stability of TYRO3 and that this process was reversible. Furthermore, we found that G-749 accelerated the RIP process and promoted TYRO degradation via the proteasomal pathway. In the current study, we found that ectodomain shedding was not inhibited when cells were co-treated with the pan-MMP inhibitors GM6001 and G-749. In contrast, when cells were co-treated with G-749 and a gamma-secretase inhibitor, Compound E, cleavage of cell membrane-anchored CTFs was completely inhibited, thereby inhibiting the production of soluble ICD (Figure 3D) (Landman and Kim, 2004; Ancot et al., 2012; Lu et al., 2017; Merilahti et al., 2017). According to previous reports, the generated soluble ICD is degraded through the proteasomal pathway or physically binds to BCL2 to amplify apoptosis-related signals (Vidal et al., 2005; Naresh et al., 2006; Merilahti and Elenius, 2019). Therefore, G-749 may contribute to tumor growth inhibition by not only inhibiting the phosphorylation of AKT and STAT3 in colon cancer, but also by generating soluble ICD. The γ-secretase complex may also cleave substrates directly without the need for ectomomain shedding (Laurent et al., 2015; Schauenburg et al., 2018; Merilahti and Elenius, 2019). Therefore, our results suggest that cleavage of TYRO3 by G-749 is mediated in a γ-secretase-dependent manner, but not by MMPs.

In the current study, we demonstrated that G-749 effectively induced apoptosis *in vitro* and *in vivo*. The flow cytometry analysis confirmed that the cell cycle was arrested in the SubG1 phase after G-749 treatment, depending on the treatment concentration, and Annexin V staining was also increased. In addition, cleaved PARP and cleaved caspase 3, which are markers of cell death, increased with G-749 treatment in a dose-dependent manner. Similar to the *in vitro* results, G-749 effectively inhibited tumor growth and induced apoptosis *in vivo*. These results suggest that G-749 exhibits significant anticancer effects in colon cancer and that G-749 can downregulate TYRO3 abnormal activity and overexpression, which may lead to the development of novel treatment strategies.

Despite the potential for excellent anticancer effects of G-749 in colon cancer, there is a potential for on-target toxicity for the use of TYRO3 as a targeting drug. According to Lee et al. (2014), G-749 showed potent inhibitory activity against MER, AXL and Aurora kinase B at the nanomolar level. MER, a proto-oncogene, is known to be involved in drug resistance and tumor growth in a variety of cancers. In addition, Aurora kinase B plays an essential role in chromosomal segregation and cytokinesis. Aurora kinase B is a potential target for anticancer drugs. According to a recent report, inhibition of AXL is known to contribute to overcoming drug resistance, activation of immune response and inhibition of metastasis in TNBC and NSCLC (Gjerdrum et al., 2010; Kim et al., 2019; Taniguchi et al., 2019; Wang et al., 2019; Yokoyama et al., 2019). TYRO3 is overexpressed in various cancers and is attracting attention as a potential cancer therapeutic target. In particular, inhibition of TYRO3 in cancers, such as bladder, colon, and ovarian cancer, effectively showed anticancer effects. Therefore, despite these limitations, the anticancer effects of G-749 targeting TYRO3 are valuable.

**FIGURE 6:**
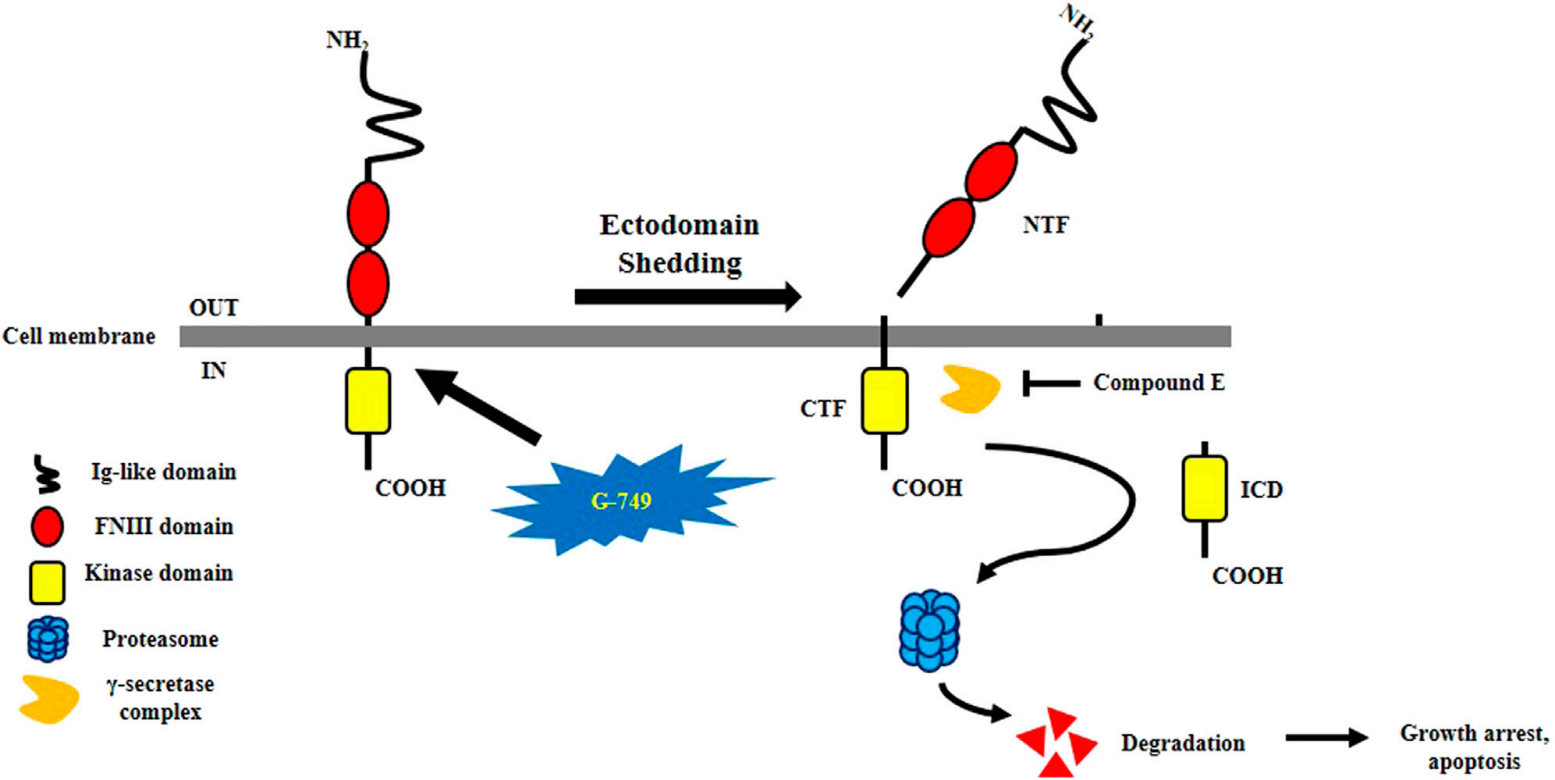
Schematization of G-749-induced TYRO3 degradation. G-749 releases the N-terminal domain of TYRO3. The intracellular C-terminal fragment is released into the cytosol by the γ-secretase complex. Compound E which is a γ-secretase inhibitor, inhibits CTF cleavage. The release of the intracellular domain was accelerated by G-749 and was later degraded via the proteasome pathway. As a result, cell growth is arrested and apoptosis is induced.

Taken together, G-749 cleaves TYRO3 via a PS-RIP-dependent pathway, and the cleaved intracellular domain is degraded via a proteasomal pathway. These results induced apoptosis in colon cancer cells (Figure 6). Although further studies are needed to determine the exact mechanism underlying G-749-induced TYRO3 fragmentation, our findings suggest that the regulation of TYRO3 protein turnover by G-749 may be a promising novel cancer treatment strategy.

## Data Availability

The original contributions presented in the study are included in the article/Supplementary Material, further inquiries can be directed to the corresponding authors.
