# Supplementary material for: G-749 Promotes Receptor Tyrosine Kinase TYRO3 Degradation and Induces Apoptosis in Both Colon Cancer Cell Lines and Xenograft Mouse Models
Source: Front Pharmacol. 2021 Oct 14;12:730241. doi: 10.3389/fphar.2021.730241 (PMC8551583; doi:10.3389/fphar.2021.730241)

Supplementary Material

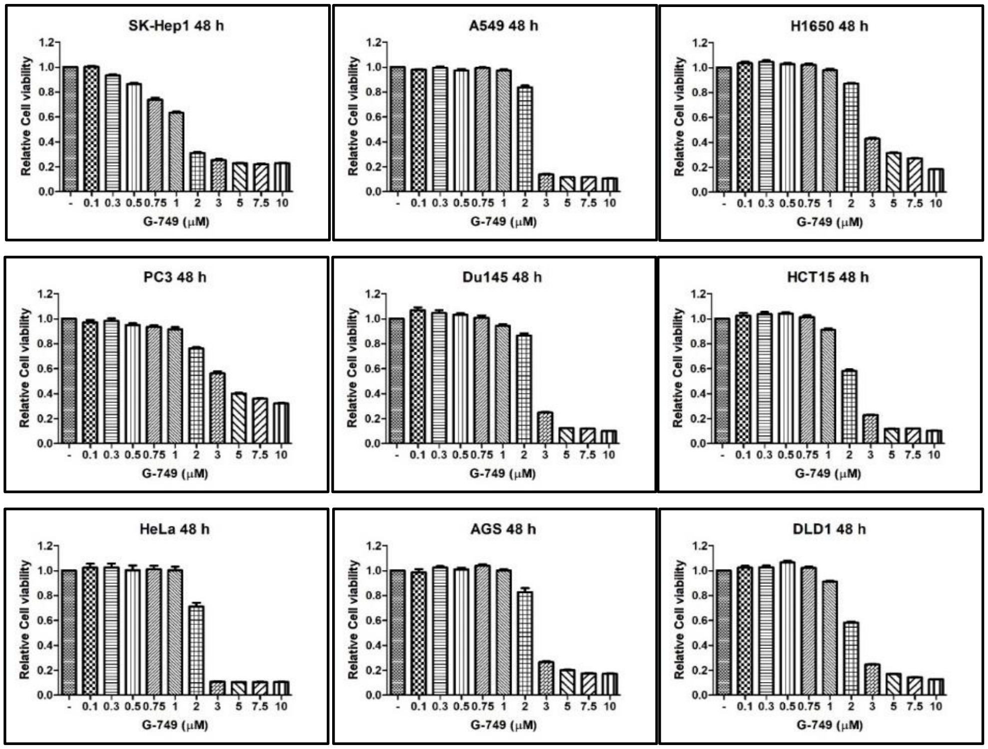

Supplementary Figure S1. Anticancer effects of G-749 in various cancer cell lines. The viability of the various cancer cell lines were measured using an MTS proliferation assay. Cells were treated with G-749 at the indicated concentration for 48 h.

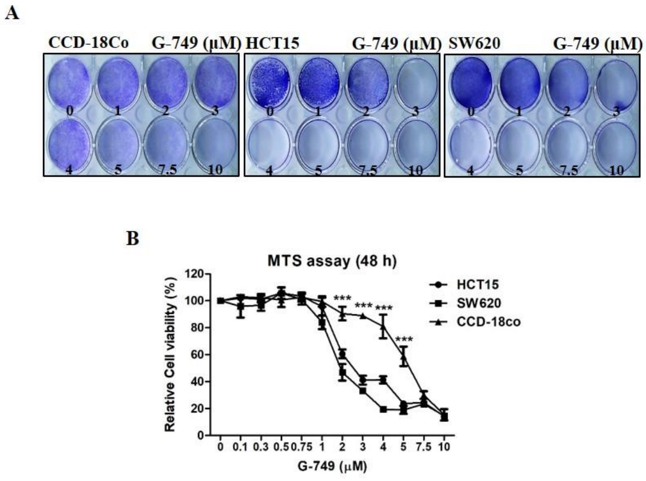

Supplementary Figure S2. Toxicity of G-749 on CCD-18co normal colon cell. (A) Cells were seeded in 12-well plates for 24 h (HCT15 and SW620) or 48 h (CCD-18co) and then treated with G-749 at the indicated concentration for 48 h. Afterwards, the cells were stained with 0.5 % crystal violet solution. (B) Cells were seeded in 96-well plates for 24 h (HCT15 and SW620) or 48 h (CCD-18co) and then treated with G-749 at the indicated concentration for 48 h. Cell viabilities were measured using an MTS assay kit.

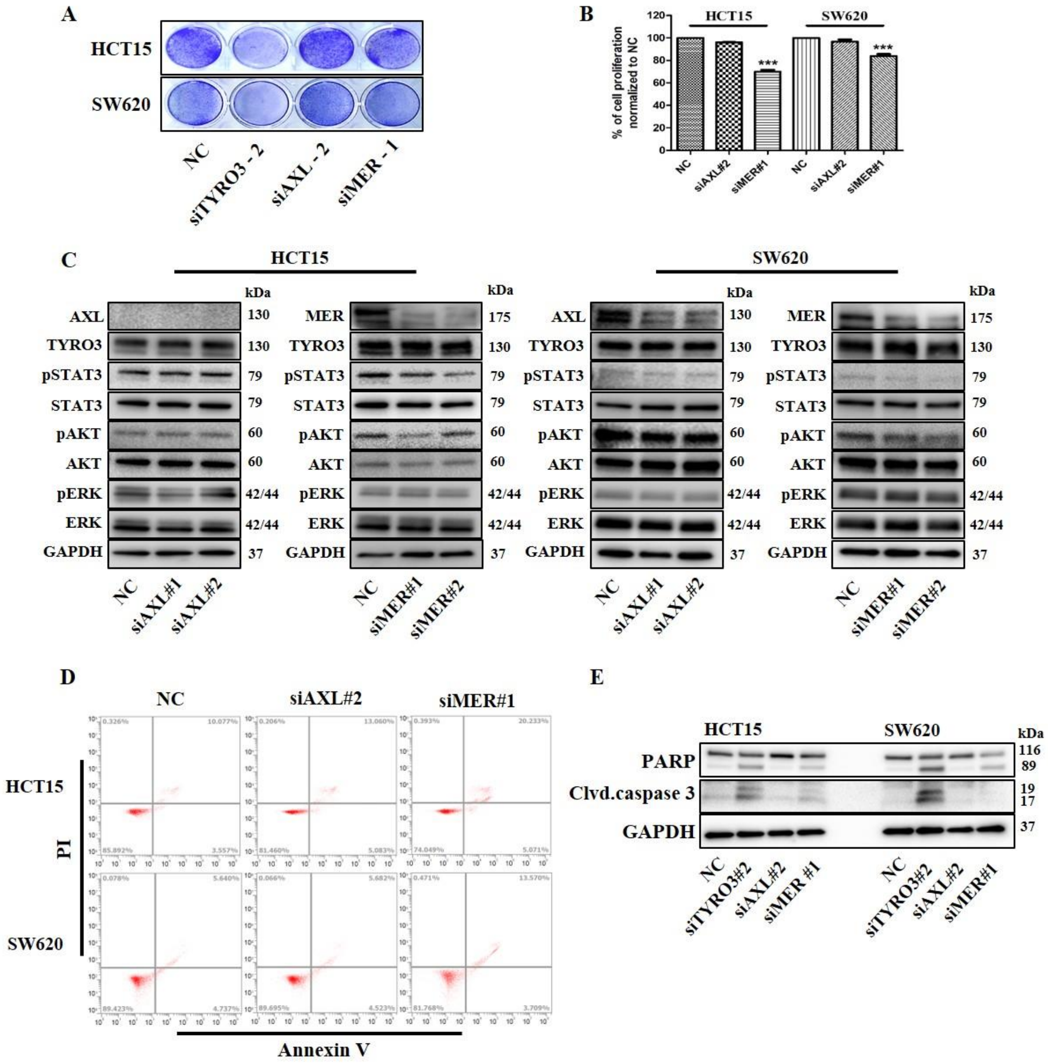

Supplementary Figure S3. Knock-down effect of AXL and MER in colon cancer cell lines. (A) Cells were transfected with TYRO3, AXL, MER and negative control (NC) siRNA for 72 h. Cells were then stained with 0.5% crystal violet. (B) Cells were incubated for 72 h after transfection with AXL, MER and NC siRNA. The viabilities of the transfected cells were measured using an MTS proliferation assay. (C) HCT15 and SW620 cells were transfected with AXL, MER and NC siRNA for 72 h. Equal amounts of cell lysates were then subjected to electrophoresis and analyzed via western blot using anti-TYRO3, -AXL, -MER, -phospho-STAT3, -STAT3, -phospho-AKT, -AKT, -phospho-ERK, -ERK, and –GAPDH antibodies. (D) HCT15 and SW620 cells were transfected with siRNA for 72 h. Apoptotic cells were stained with propidium iodide/Annexin V and then analyzed by flow cytometry. (E) Cells were transfected with TYRO3, AXL, MER and NC siRNA for 72 h and then analyzed via western blot using anti-TYRO3, -PARP, -cleaved caspase 3 and –GAPDH antibodies. Data are presented as means ± standard deviations (SD). Data were analyzed using one-way ANOVA with Tukey's multiple comparison post-hoc analysis; ****$p < 0.0001$, ***$p < 0.001$, **$p < 0.01$, *$p < 0.05$

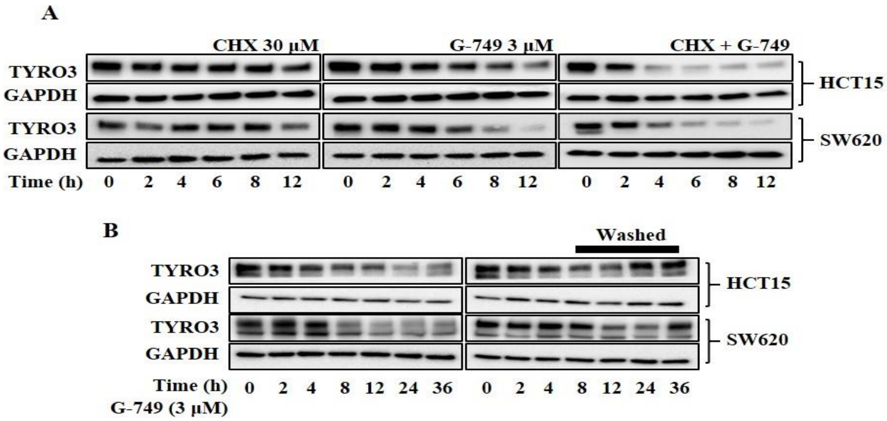

Supplementary Figure S4. G-749-induced TYRO3 protein turnover in colon cancer cells. (A) Cells were treated with CHX (30 µM) and G-749 (3 µM) for the indicated times. Cell lysates were subjected to electrophoresis and analyzed by western blot using anti-TYRO3 and GAPDH antibodies. GAPDH was used as a loading control. (B) G-749-induced reduction of TYRO3 is reversible. HCT15 and SW620 cells were treated with G-749 for 8 h and then washed with fresh medium or not. Cells were harvested at the indicated time periods. The cultured cells were lysed and analyzed by western blot with anti-TYRO3 antibodies.

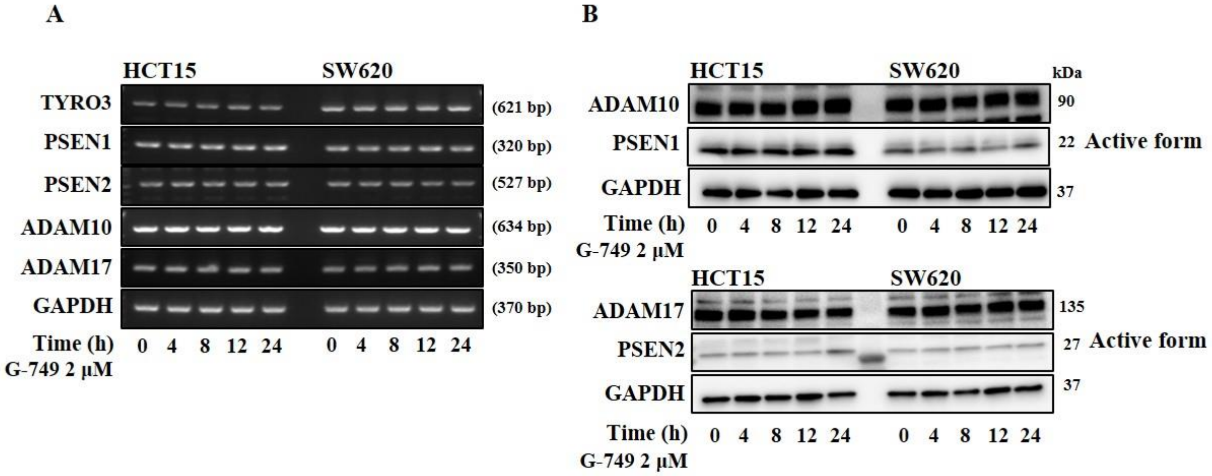

Supplementary Figure S5. Effect of G-749 treatment on ADAM10, ADAM17, PSEN1 and PSEN2 in colon cancer cells. (A) RT-PCR analyses of cells treated with G-749 (2 µM) for the indicated times and harvested at the indicated time periods. GAPDH was used as a loading control. (B) Western blot analyses of cells treated with G-749 (2 µM) for the indicated times and harvested at the indicated time periods. Equal amounts of cell lysates were subjected to electrophoresis and analyzed using antibodies against ADAM10, ADAM17, PSEN1 and PSEN2. GAPDH was used as a loading control.

Supplementary Table S1. Antibodies used in this paper

Target	Source & Cat.No	ratio	Target	Source & Cat.No	ratio
TYRO3 (C terminal)	CST #5585	1:1000	Phospho ERK	CST #4370	1:1000
MERTK	CST #4319	1:1000	FLT3	CST #3462	1:1000
AXL	CST #4566	1:1000	PE conjugated TYRO3 (N terminal)	R&D systems FAB859P	1:100
GAPDH	Santa cruz Sc-47724	1:2000	PE-conjugated Mouse IgG_1	R&D systems IC002P	1:100
AKT	CST #9272	1:1000	β-actin	Santa cruz Sc-47778	1:2000
ERK	CST #4695	1:2000	Phospho TYRO3/AXL/MERTK	CST #44463	1:1000
Caspase3	CST #9662	1:1000	Phospho AXL	CST #5724	1:1000
Cleaved caspase3	CST #9661	1:1000	Phospho STAT3	CST #9145	1:1000
PARP	CST #9542	1:1000	Phospho AKT	CST #9271	1:1000
ADAM10	CST #14194	1:1000	Ki-67	CST #9129	1:200
ADAM17	CST #3976	1:1000	STAT3	CST #12640	1:1000
PSEN-1	CST #5643	1:1000	Alexa Fluor 488 Goat anti-Rabbit IgG secondary	Invitrogen A11034	1:500
PSEN-2	CST #2192	1:1000	Phospho MERTK	Ab14921	1:1000
Phospho MERTK	Phosphosoutions p-186-749	1:1000	Phospho MERTK	FabGennix PMKT-140AP	1:1000

Supplementary Table S2. siRNA used in this paper

Target	Sense sequence (5`-3`)	Anti-sense sequence (5`-3`)	Source
TYRO3#1	CACAGUGGAGCCAAAAGAUTT	AUCUUUUGGCUCCACUGUGTT	Bioneer (Daejeon, Korea)
TYRO3#2	GCUGUUAGUUGCUGUUUAATT	UUAAACAGCAACUAACAGCTT	doi.org/10.1038/s41416-019-0397-6
AXL#1	GGGUGGAGGUUAUCCUGAATT	UUCAGGAUAACCUCCACCCTC	doi.org/10.1038/s41416-019-0397-6
AXL#2	GACUGUCUGGAUGGACUGUTT	ACAGUCCAUCCAGACAGUCTT	Bioneer (Daejeon, Korea)
MER#1	GGAUGAAGCCUCCGACUAATT	UUAGUCGGAGGCUUCAUCCAT	doi.org/10.1038/s41416-019-0397-6
MER#2	CUCAUGAAGGACGGUACAUTT	AUGUACCGUCCUUCAUGAGTT	Bioneer (Daejeon, Korea)
Negative Control	CCUCGUGCCGUUCCAUCAGGUAGUU	CUACCUGAUGGAACGGCACGAGGUU	Genolution (Seoul, Korea)

Supplementary Table S3. PCR primers used in this paper

Genes	Annealing temperature (℃)	Size (bp)	Sense (5’ – 3’)	Anti-sense (5’ – 3’)
Human TYRO3	61	621	TGACCATCCACACGTGGCCAA	AGACAAGTAAAGCTCGGGCGC
Human ADAM10	60	634	GAGAAGTGTCGGGATGATTC	GGGGTTGCTGAATGGGCTGT
Human ADAM17	60	350	GACCTTTCTGGCCGCTGTGT	GCAGAATCCATGCTGCTCAG
Human PSEN1	56	320	GACTCCAGCAGGCATATCTC	GTCTTGTGACTCCCTTTCTG
Human PSEN2	62	527	CAGTGGGCATGGTGTGCATC	CAGCGTGGTATTCCAGTCCC
Human GAPDH	58	370	TTTGGTCGTATTGGGCGCCTG	CCATGACGAACATGGGGGCAT

RT-PCR raw data

Figure 1C

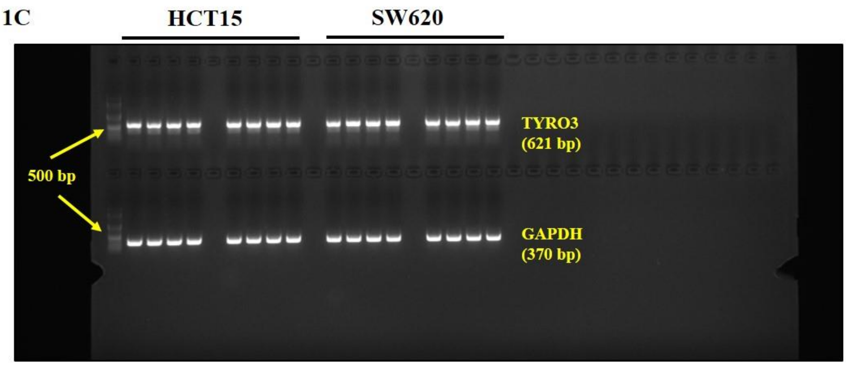

Supplementary Figure S5A

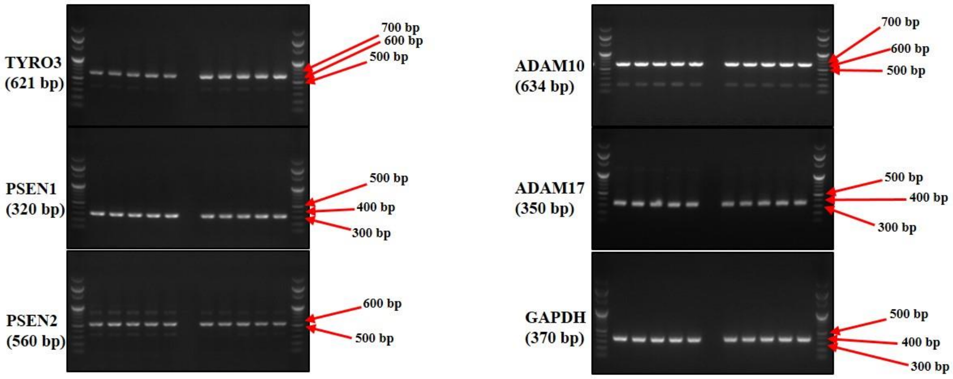

Western blot raw data

Figure 1B

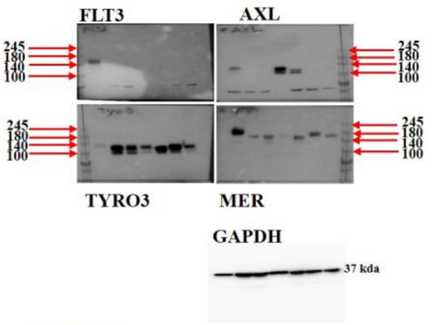

Figure 1C

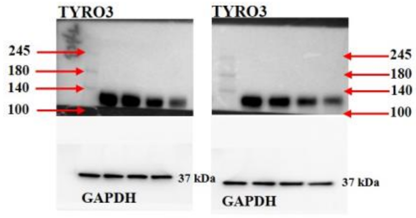

Figure 1E

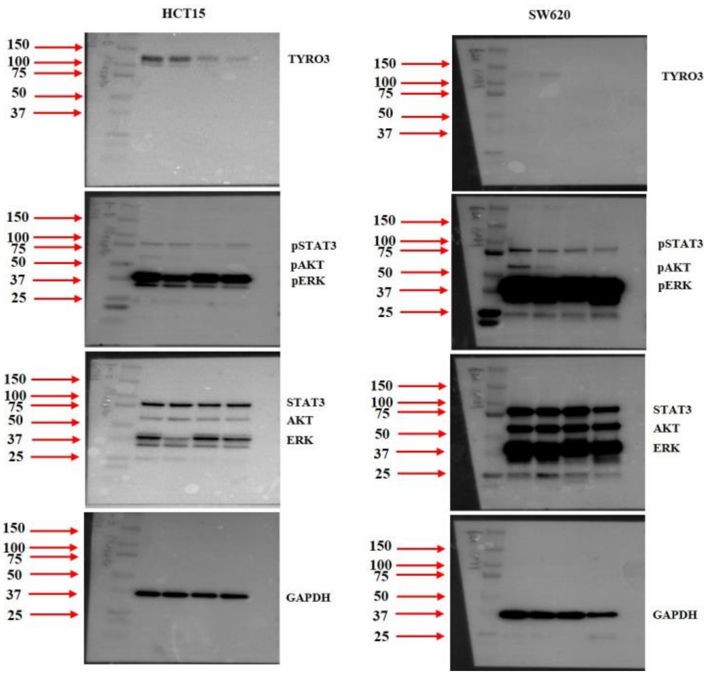

Figure 2C

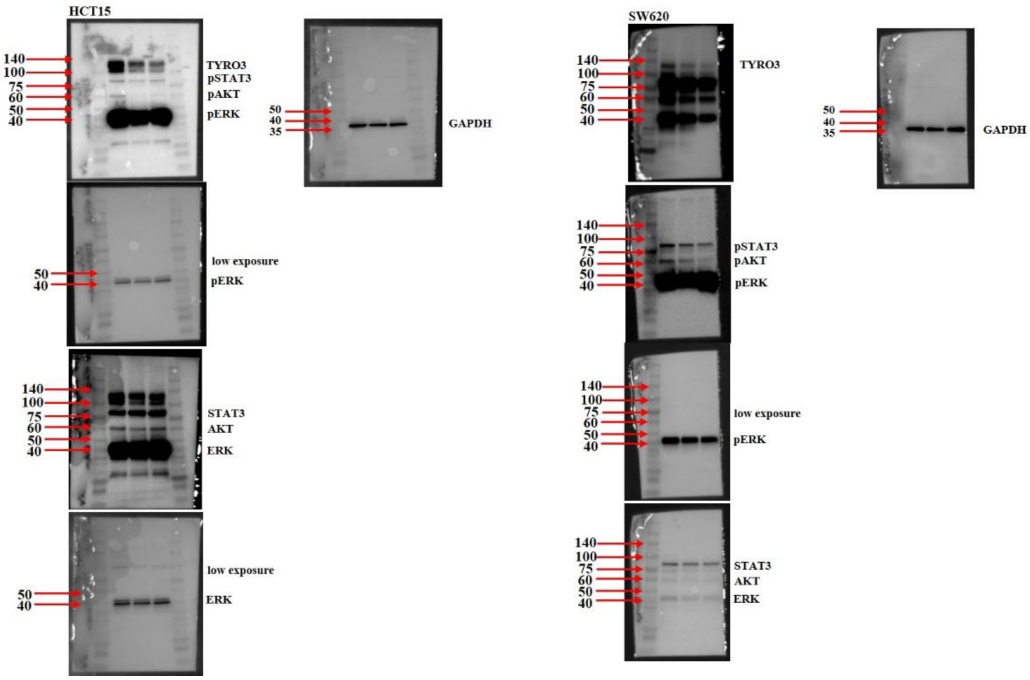

Figure 2F

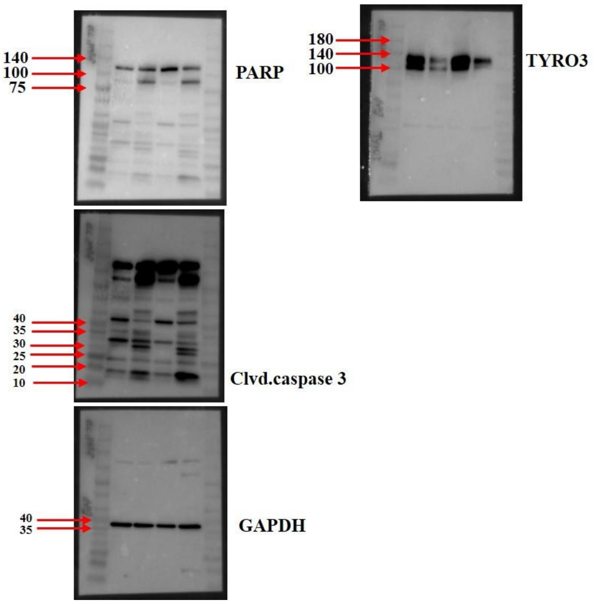

Figure 3C

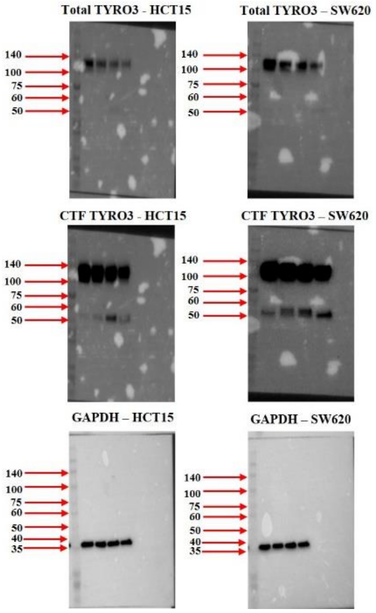

Figure 3D

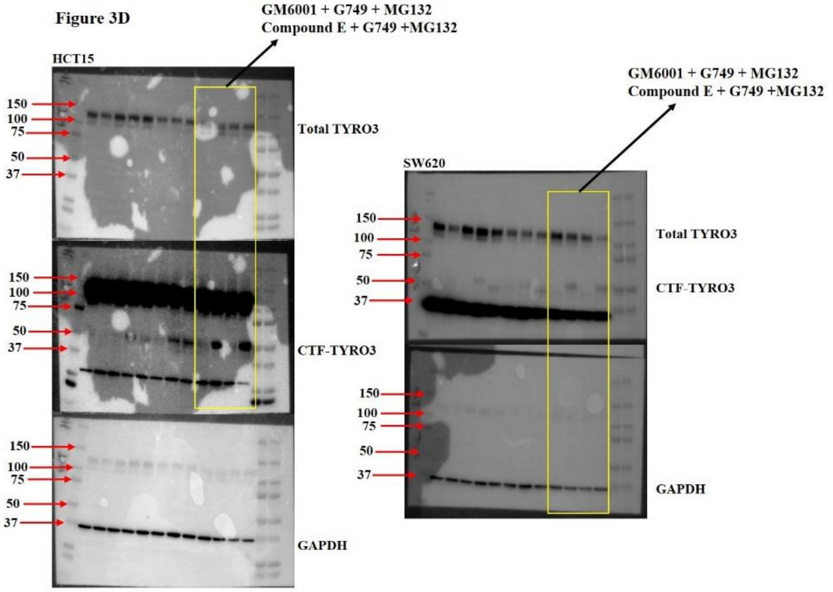

Figure 3E

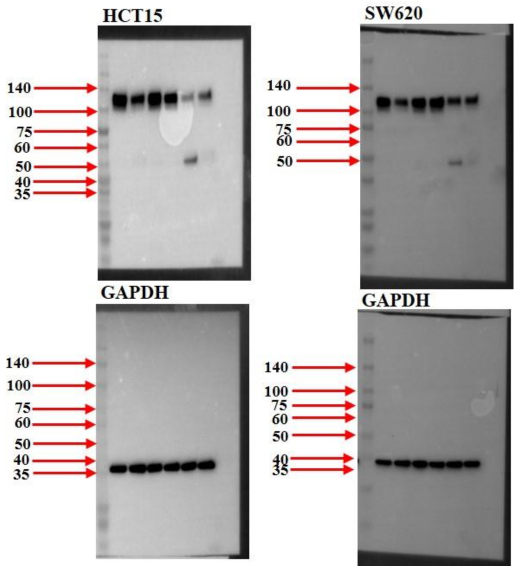

Figure 4C

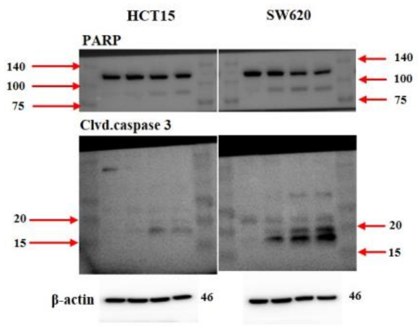

Figure 4D

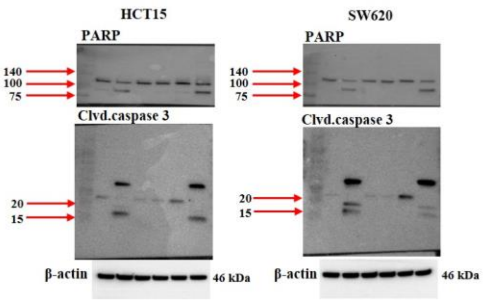

Figure 5D

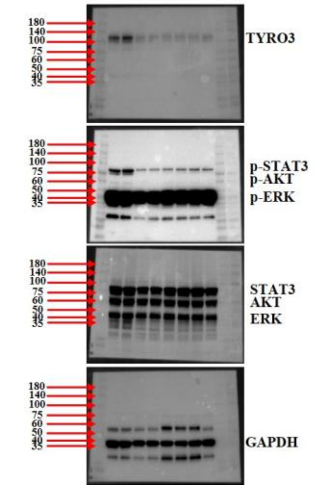

Supplementray raw data

Supplementary Figure S3C

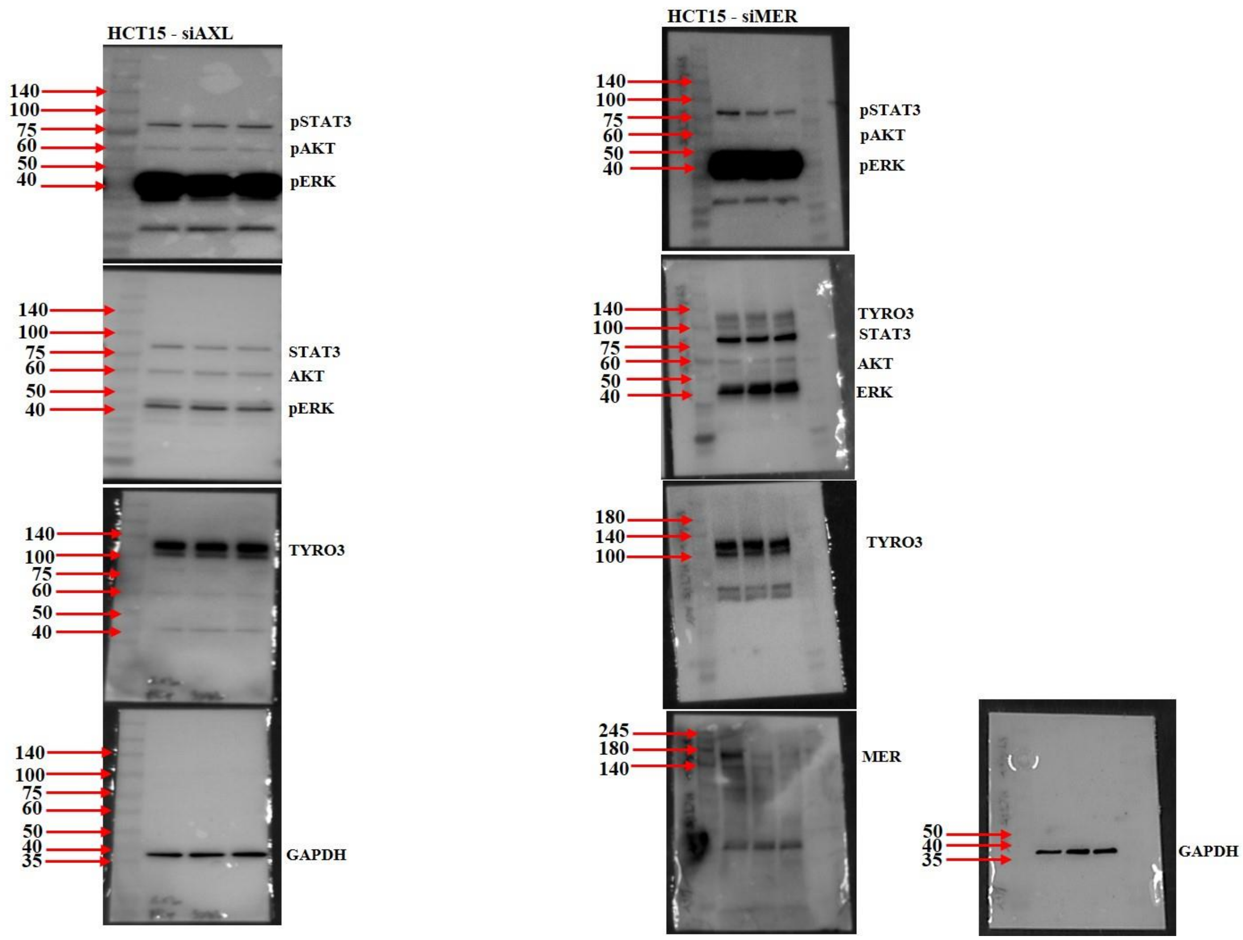

Supplementary Figure S3C

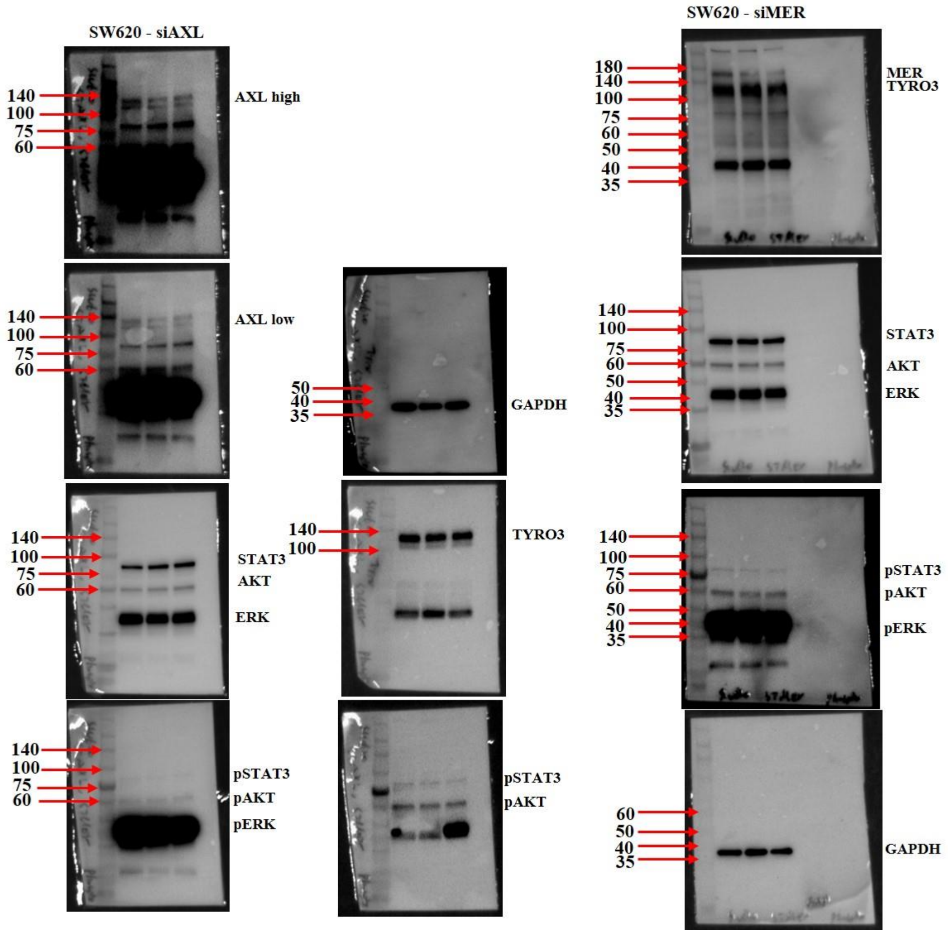

Figure S3E

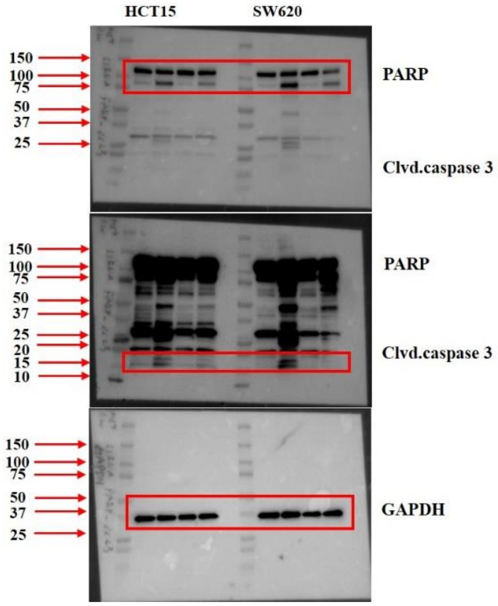

Supplementary Figure S4

Figure S4A

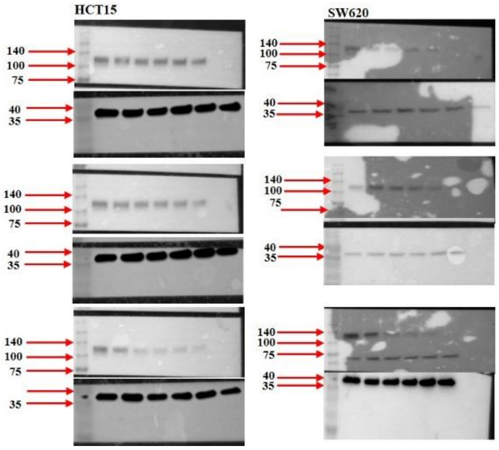

Figure S4B

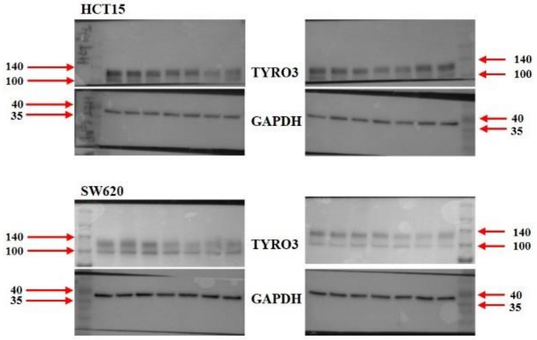

Figure S5B

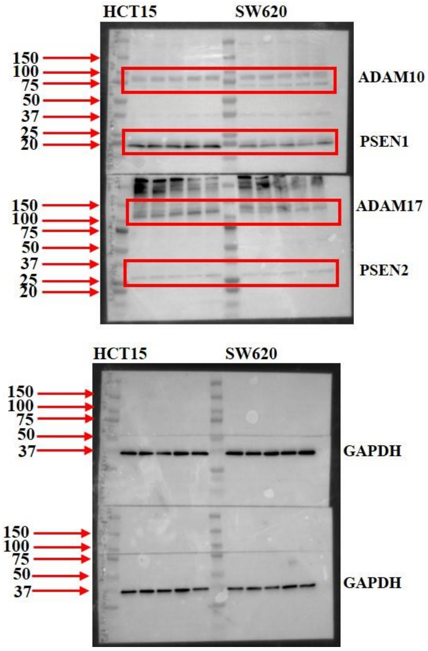

Supplement: Supplementary file 1 [file DataSheet1.pdf]
